# Crystal Structure of a Phospholipase D from the Plant-Associated Bacteria *Serratia plymuthica* Strain AS9 Reveals a Unique Arrangement of Catalytic Pocket

**DOI:** 10.3390/ijms22063219

**Published:** 2021-03-22

**Authors:** Fanghua Wang, Siyu Liu, Xuejing Mao, Ruiguo Cui, Bo Yang, Yonghua Wang

**Affiliations:** 1 School of Food Science and Engineering, South China University of Technology, Guangzhou 510640, China; wangfanghua@scut.edu.cn (F.W.); liusiyu1234562021@163.com (S.L.); maoxuejing@mail.scut.edu.cn (X.M.); ruigc_scut@163.com (R.C.); 2 School of Bioscience and Bioengineering, South China University of Technology, Guangzhou 510006, China; yangbo@scut.edu.cn

**Keywords:** phospholipase D, *Serratia plymuthica* strain AS9, bioinformatics, crystal structure

## Abstract

Phospholipases D (PLDs) play important roles in different organisms and in vitro phospholipid modifications, which attract strong interests for investigation. However, the lack of PLD structural information has seriously hampered both the understanding of their structure–function relationships and the structure-based bioengineering of this enzyme. Herein, we presented the crystal structure of a PLD from the plant-associated bacteria *Serratia plymuthica* strain AS9 (SpPLD) at a resolution of 1.79 Å. Two classical HxKxxxxD (HKD) motifs were found in SpPLD and have shown high structural consistence with several PLDs in the same family. While comparing the structure of SpPLD with the previous resolved PLDs from the same family, several unique conformations on the C-terminus of the HKD motif were demonstrated to participate in the arrangement of the catalytic pocket of SpPLD. In SpPLD, an extented loop conformation between β9 and α9 (aa228–246) was found. Moreover, electrostatic surface potential showed that this loop region in SpPLD was positively charged while the corresponding loops in the two *Streptomyces* originated PLDs (PDB ID: 1F0I, 2ZE4/2ZE9) were neutral. The shortened loop between α10 and α11 (aa272–275) made the SpPLD unable to form the gate-like structure which existed specically in the two *Streptomyces* originated PLDs (PDB ID: 1F0I, 2ZE4/2ZE9) and functioned to stabilize the substrates. In contrast, the shortened loop conformation at this corresponding segment was more alike to several nucleases (Nuc, Zuc, mZuc, NucT) within the same family. Moreover, the loop composition between β11 and β12 was also different from the two *Streptomyces* originated PLDs (PDB ID: 1F0I, 2ZE4/2ZE9), which formed the entrance of the catalytic pocket and were closely related to substrate recognition. So far, SpPLD was the only structurally characterized PLD enzyme from *Serratia*. The structural information derived here not only helps for the understanding of the biological function of this enzyme in plant protection, but also helps for the understanding of the rational design of the mutant, with potential application in phospholipid modification.

## 1. Introduction

Phospholipases D (PLDs, EC 3.1.4.4) belong to the phospholipase superfamily and they are ubiquitously distributed in various organisms including mammals, plants and microorganisms [1]. PLDs catalyze two kinds of reactions: (1) hydrolysis reaction, in which PLDs cleave the terminal phosphodiester bonds of phospholipids to produce phospholipid acids (PA) and the free alcohol moieties, and (2) transphosphatidylation reaction, in which PLDs transfer phosphatidic acids to acceptor alcohol moieties to form new phospholipids [2]. Since their first discovery in carrots and cabbages leaves in 1940s [3,4], PLDs have gained much attention both in their biological functions and in their applications for industial phospholipid modification, based on their unique transphosphatidylation activities. In the aspect of protein structure, members in the PLD superfamily contain one or two highly conserved HxKxxxxDxxxxxxGG/S (HKD) motifs, which were assumed to act as active centers [5]. According to the PFam database (http://pfam.xfam.org/ (accessed on 5 February 2021)), currently there are 58,157 protein sequences from 7682 species in the PLD superfamily (Clan: CL0479) [6]. This superfamily contains 11 subfamilies and most of the reported PLDs belong to the PLDc 2 family (28,771 sequences from 6957 species). However, until now, only 9 PLD structures (not including the mutant or complex) from 8 species have been reported within this family (Table 1). Except for the Nuc (PDB ID: 1BYS/1BYR) [7], Zuc (PDB ID: 4GEL) [8], mZuc (PDB ID: 4GGJ and 4GGK) [9], Bfi1(PDB ID: 2C1L) [10], NucT(PDB ID: 6EHI) [11] that were actually functioned as nucleases to hydrolyze DNA, only the *Streptomyces* sp. PLD and the *Homo sapiens* PLD (hPLD1 and hPLD2) were the true PLDs that accepted phoshplipids as substrates. The structural information of PLDs is important for the understanding of the enzyme catalytic mechanism and provides guidance for enzyme engineering. The first authentic PLD tertiary structure resolved in 2000 was the PLD from *Streptomyces sp*. PMF [12]. Based on this structure, a two-step reaction mechanism was elucidated [13]. In 2007, crystal structures of the apo *Streptomyces antibioticus* PLD (SaPLD) and the complex with phosphatidylcholine have been resolved. Based on these structures, various SaPLD mutants with enhanced thermostability and selectivity have been designed and discovered, which further promoted the industrial application of this enzyme [14,15,16]. In 2019, two catalytic domains of hPLD1 and hPLD2 which both contained small-molecule inhibitors were resolved. Characterization of the enzyme architectures, understanding the binding modes of these inhibitors and identification of key residues that provide isoenzyme selectivity to make the design and optimization of drugs that target PLDs more efficient [17,18]. Although some achievements have been made by comparing to the large amount of sequence information in this family, the lack of structural information has hindered the in-depth understanding of the structure–function relationship of PLDs.

In the present study, the crystal structure of a PLD from the plant-associated bacteria *Serratia plymuthica* strain AS9 was solved at 1.79 Angstrom (Å) resolution. Furthermore, by comparing the structure of SpPLD with the previously resolved PLD structures from the same family, a unique arrangement of the catalytic pocket was demonstrated. So far, this is the only structurally characterized PLD enzyme from *Serratia*. These results not only refined the phylogenetic and structural information of the PLD databases, but also laid a foundation to understand the structure–function relationship of this enzyme.

## 2. Results

### 2.1. Bioinformatic Analysis

**Figure 1:**
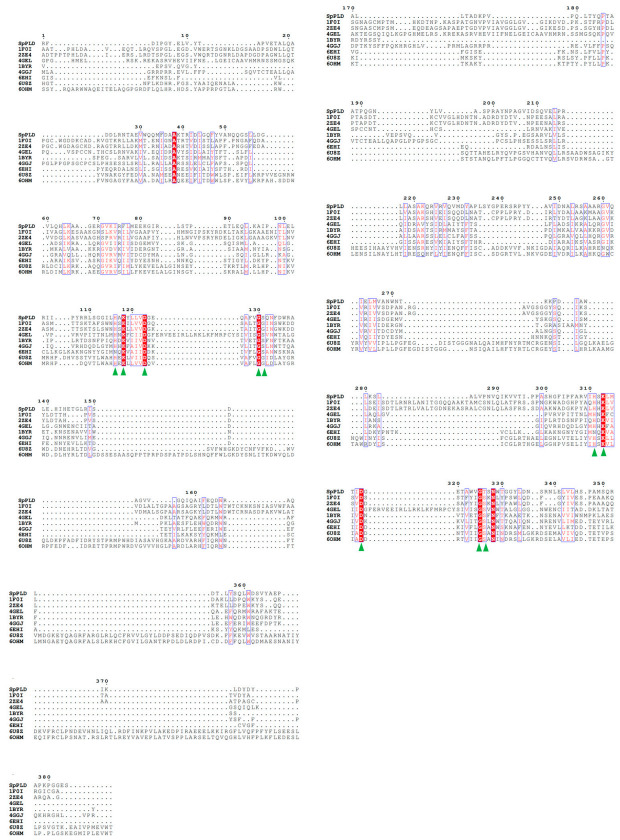
Structure-based amino acid sequence alignment of the mature SpPLD with several other PLDs in the PLDc 2 family. Residues in the conserved HKD motif of the PLD superfamily were indicated with green triangles. For the NucT from *Helicobacter pylori* (PDB ID: 6EHI), two histidines in the HKD motifs were mutanted to glutamine to obtain enzyme structure in the inactive form.

**Figure 2:**
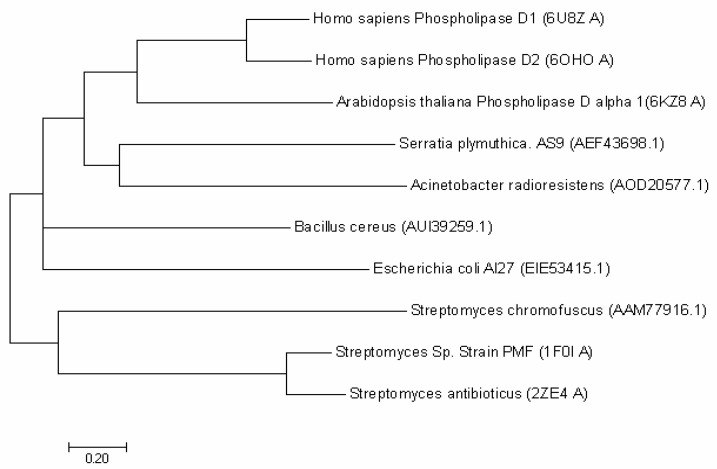
Phylogenetic analysis of SpPLD with other PLDs. Phylogenetic analysis with neighbor-joining (NJ) was conducted by using MEGA 7.0 software (Hachioji, Tokyo, Japan). Different PLD protein sequeces used for present analysis were shown in the bracket.

The full-length SpPLD contains 416 amino acids and the first 32 amino acids (MPNGRQPRRPLGRLYTALWGSCLLMLSQSAAA) were predicted to be the signal peptide. No potential transmembrane domain region was found in the protein sequence analysis, indicating that the enzyme was an extracellular protein. Structure-based amino acid sequence alignment of the mature SpPLD with several other PLDs in the PLDc 2 family was shown in Figure 1. Two highly conserved motifs (HxKxxxxD) comprising the the putative catalytic residues His116 and His311 were found in SpPLD (Figure 1). Two GG/S motifs downstream of the corresponding HKD motifs also existed in the SpPLD. However, the first GG/S motif was composed of GS which also appreared in the nulceases Zuc (PDB ID: 4GEL), NucT (PDB ID: 6EHI), Nuc (PDB ID: 1BYR) and mZuc (PDB ID:4GGJ) within the same family. In constrast, in PLDPMF (1F0I), SaPLD (2ZE4) and two *Homo sapiens* PLDs (hPLD1 PDB ID: 6U8Z and hPLD2 PDB ID: 6OHM), the corresponding GG/S motif was composed of GG but not GS. The second GG/S motif is different between all the PLDs in the same family while the SpPLD has GT but not GS. Protein sequence identity between SpPLD, PLDPMF and SaPLD is quite low (less than 20%), and there was no significant sequence similarities from one to another except for the already mentioned conserved HKD motifs (Figure 1). Phylogenetic analysis of PLDs showed that SpPLD was highly related to the PLD from *Acinetobacter radioresistens* (GenBank: AOD20577.1), indicating that they were homologous in the evolutionary process (Figure 2). The highly conserved motifs present in the primary structure and the high phylogenetic relationship strongly indicated that SpPLD was a member of the PLD superfamily.

### 2.2. Expression and Purification of the Mature SpPLD

**Figure 3:**
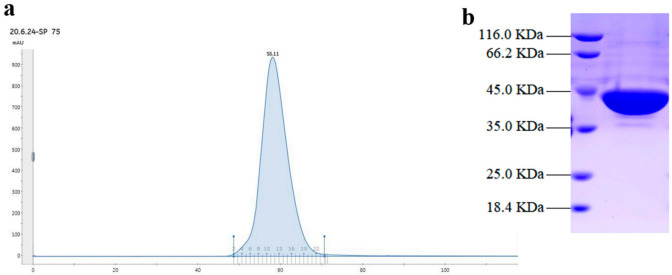
Gel filtration chromatography (**a**) and sodium dodecyl sulphate-polyacrylamide gel electrophoresis (SDS-PAGE) analysis of the purified SpPLD (**b**).

Gel filtration chromatography showed a single and symmetrical peak of the target protein, which indicated the homogeneity of the recombinant SpPLD (Figure 3a). SDS-PAGE revealed that the recombinant SpPLD with 8×His-tag was approximately 44 kDa, which matched the theoretical molecular weight (43.7 kDa) (Figure 3 b).

### 2.3. X-ray Diffraction Results

Crystal structure of the ligand-free SpPLD was obtained at 1.79 Å resolution by the single-wavelength anomalous diffraction (SAD) method. It belonged to a P2_1_2_1_2_1_ space group with unit-cell parameters of a = 78.78 Å, b = 97.57 Å, c = 117.03 Å, α = 90 Å, β = 90 Å, γ = 90 Å. Two molecules were found in one asymmetric unit, and the root-mean square deviation (RMSD) value of 307 backbone Cα between the two molecules was 0.126 Å. 759 water molecules in total were found in one asymmetric unit. After the repeating modification and optimization, R_factor_ and R_free_ values of the final model were 16.95% and 18.48%, respectively (Table 2).

### 2.4. Overall Structure

**Figure 4:**
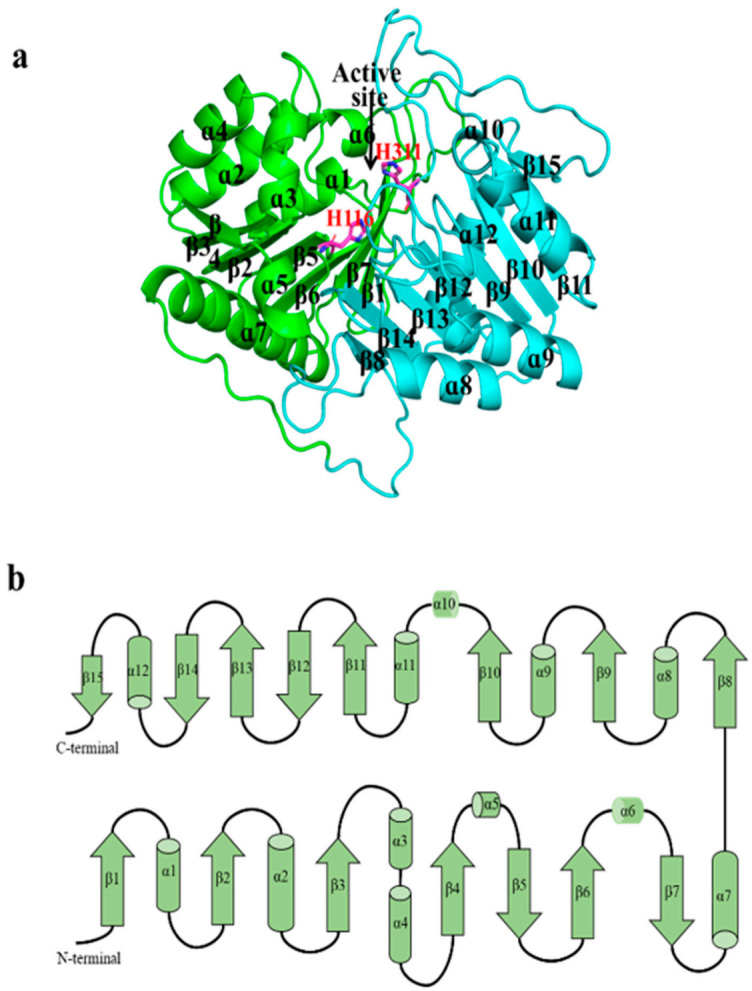
(**a**) The overall structure of SpPLD. SpPLD was composed of two domains colored with green and light blue, respectively. The active site histidines His116 and H311 were located at the protein active site. The N-terminal domain was composed of 7 α helixs and 7 β sheets and the C-terminal domain was composed by 5 α helixs and 8 β-sheets. (**b**) Overall topology of SpPLD.

The overall structure of SpPLD displayed a β-α-β-α-β- sandwich fold. SpPLD contained in total 15 β-strands, 12 α-helices and a series of ordered loops that combined to form a single asymmetric globular structure (Figure 4a). The structure was composed of two tightly interacting HKD domains with slightly different topology (Figure 4b). The N-terminal domain was composed of 7 β-sheets and 7 α-helices, with 2 β-sheets (β5 and β7) antiparallel to the other sheets (β1–β7). The C-terminal domain was composed of 8 β-sheets and 5 α-helices. Among them, 3 β-sheets (β12, β14 and β15) were antiparallel to the others (β8–β15). The two HKD domains self-dimerized in close proximity to form a single active site with the catalytic histidine (H116, H311) and lysine residues (K118, K313). The dimensions of SpPLD in terms of gyration radii was defined to be 19.76 Å.

### 2.5. Structural Comparison

#### 2.5.1. Overall Structure Comparion

**Figure 5:**
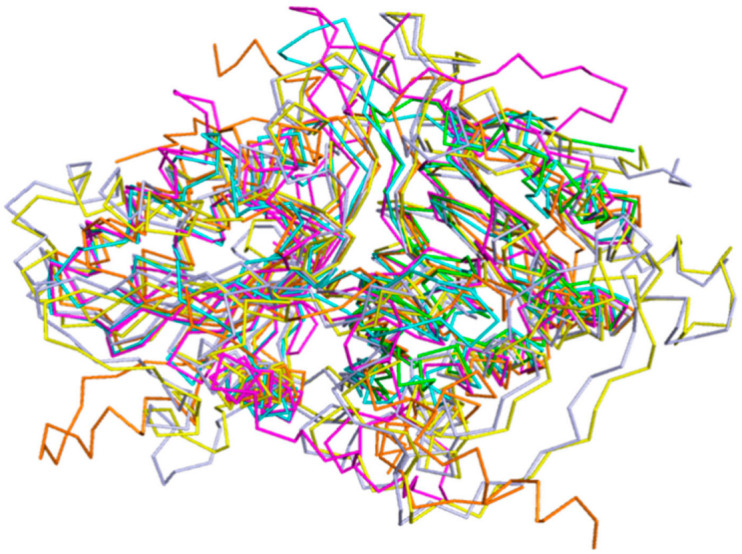
Stereo-view of the superimposition of SpPLD (magenta, PDB ID: 7E0M), *Streptomyces* sp. PMF PLD (yellow, PDB ID: 1F0I), SaPLD (light blue, PDB ID: 2ZE4), Zuc (orange, PDB ID: 4GEL), NucT(cyans, PDB ID: 6EHI) and Nuc (green, PDB ID: 1BYR).

Analysis on the DALI online server indicated that the phosphatidyl serine synthase (PDB entry: 3HSI, Z-score of 28) showed the highest similarity to SpPLD and the root-mean-square deviation (RMSD) between the 318 Cα atoms of these two structures was 3.1 Å. This phosphatidyl serine synthase belonged to the PLDc (PF00614) family according to the PFam database. The second most similar enzyme to SpPLD was the *Escherichia coli* polyphosphate kinase (EcPPK, 1XDO), with the Z-score of 26.6 and RMSD between the 293 Cα atoms of the two structures 3.3 Å. The EcPPK belonged to the PP_kinase_C family (PF13090). Within the family of PLDc 2, structures of 2ZE9 (Z-score of 26.0, RMSD 3.2 Å), 6EHI (Z-score of 19.7, RMSD 2.1 Å), 1BYR (Z-score of 19.2, RMSD 2.1 Å), 4GEL (Z-score of 15.7, RMSD 2.6 Å) were also found similar to SpPLD. Structural comparison of SpPLD and the PLDPMF (PDB code: 1F0I) showed that in total 225 Cα atoms from each molecule can be well-superimposed, with a RMSD of 3.7 Å. Except for the loop region, the main secondary structure fold and the topology of SpPLD were similar to the other PLDs within the same family (Figure 5). The main differences of the loop conformation are illustrated below.

#### 2.5.2. Conserved HKD Motifs in the Catalytic Pocket of SpPLD

**Figure 6:**
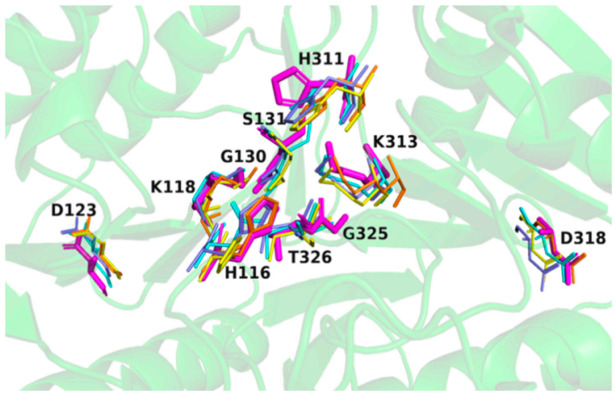
Two classical HxKxxxxD (HKD) motifs were found in SpPLD and showed high structural consistence with several PLDs within the same family. SpPLD (magentas, PDB ID: 7E0M), *Streptomyces sp.* PMF PLD (yellow, PDB ID: 1F0I), SaPLD (light blue, PDB ID: 2ZE4), Zuc (orange, PDB ID: 4GEL), NucT(cyans, PDB ID: 6EHI).

Structural superimposition of SpPLD with other PLDs in the same family showed that HKD motifs were highly conserved within this PLD family (Figure 6). The two catalytic histidine residues of the HKD motifs in SpPLD were both located at the interface of the two core β-sheets at the distance of 6.7 Å. The N_1_ nitrogen atoms at the side chains of H116 and H311 formed hydrogen bonds to residues N339 and K313, respectively.

#### 2.5.3. Unique Arrangement in the Catalytic Pocket of SpPLD

**Figure 7:**
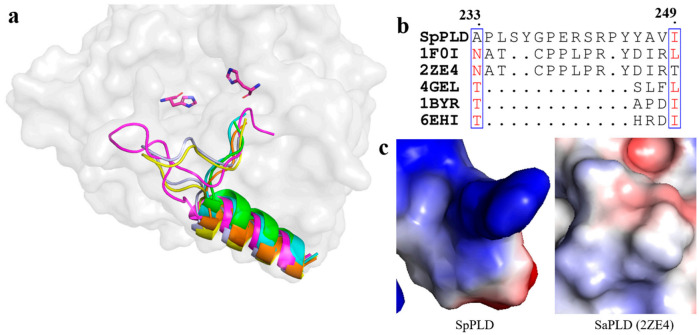
(**a**) Superimpostion and comparision the loop between β9 and α9 with other PLDs. SpPLD (magentas, PDB ID: 7E0M), *Streptomyces* sp. PMF PLD (yellow, PDB ID: 1F0I), SaPLD (light blue, PDB ID: 2ZE4), Zuc (orange, PDB ID: 4GEL), NucT (cyans, PDB ID: 6EHI), Nuc (green, PDB ID: 1BYR). Two catalytic sites of SpPLD were shown in stick. (**b**) Sequence alignment of the amino acids that composed the loop between β9 and α9. (**c**) Electrostatic surface potential profile of the loop between β9 and α9 in SpPLD and the corresponding loop in SaPLD (2ZE4).

As mentioned before, several loops also participated in the arrangement of the catalytic pocket of SpPLD except for the two HKD motifs. The arangement of several loops were found distinct from other PLDs based on the structural superimposition of SpPLD and other PLDs within the same family. An extented loop was found between α9 and β9, which was distinct from other PLDs (Figure 7a). This loop had an extra of three amino acids comparing to the 1F0I and 2ZE4 (Figure 7b). Moreover, the electrostatic surface potential of SpPLD showed that this loop region was positively charged with a net charge of +1. In constrast, the total net charge of the corresponding loop region in SaPLD (2ZE4) was 0 and was actually hydrohobic. Moreover, the corresponding loop was shorter for the several nucleases (4ZEL, 1BYR and 6EHI) (Figure 7).

#### 2.5.4. An Extent Loop Was Found between α9 and β9 That Distinct from Other PLDs

**Figure 8:**
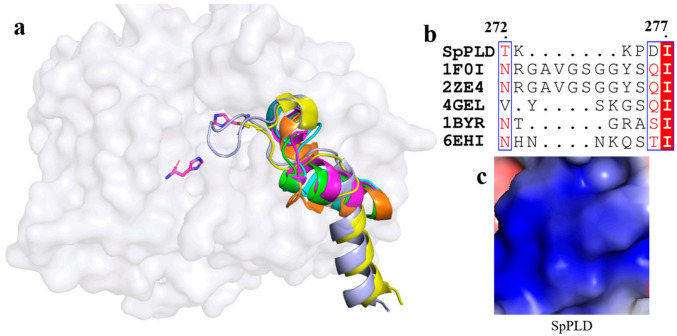
(**a**) Superimpostion and comparision the loop between α10 and α11 with other PLDs. SpPLD (magentas, PDB ID: 7E0M), *Streptomyces* sp. PMF PLD (yellow, PDB ID: 1F0I), SaPLD (light blue, PDB ID: 2ZE4), Zuc (orange, PDB ID: 4GEL), NucT(cyans, PDB ID: 6EHI), Nuc (green, PDB ID: 1BYR). Two catalytic sites of SpPLD were shown in stick. (**b**) Sequence alignment of the amino acids that composed the loop between α10 and α11 in SpPLD with other PLDs. (**c**) Electrostatic surface potential profile of the loop between α10 and α11 of SpPLD.

On the other hand, a short loop existed between α10 and α11 in SpPLD. Extented loops were found between the corresponding α10 and α11 in PLDPMF (PDB ID: 1F0I) and SaPLD (PDB ID: 2ZE4), while similar short loops existed in Nuc (1BYR) and Zuc (4GEL) between α10 and α11 (Figure 8a). The electrostatic surface potential showed that the loop in SaPLD was positively charged (Figure 8c) because of the presence of arginine and lysine (Figure 8b).

A short loop existed between β11 and β12.

**Figure 9:**
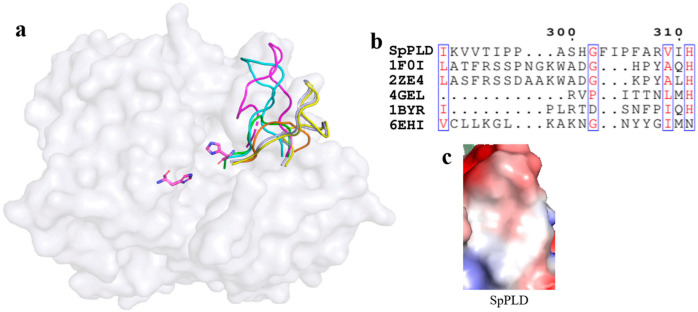
(**a**) Superimpostion and comparision the loop between β11 and β12 with other PLDs. SpPLD (magentas, PDB ID: 7E0M), *Streptomyces sp*. PMF PLD (yellow, PDB ID: 1F0I), SaPLD (light blue, PDB ID: 2ZE4), Zuc (orange, PDB ID: 4GEL), NucT(cyans, PDB ID: 6EHI), Nuc (green, PDB ID: 1BYR). Two catalytic sites of SpPLD were shown in stick. (**b**) Sequence alignment of the amino acids that composed the loop between β11 and β12 in SpPLD with other PLDs. (**c**) Electrostatic surface potential profile of the loop between β11 and β12 of SpPLD.

While superimposing and comparing the loop with other PLDs, a slightly shorter loop between β11 and β12 of SpPLD was found, compared to the corresponding loops in PLDPMF (PDB ID: 1F0I) and SaPLD (PDB ID: 2ZE4) (Figure 9a). However, this loop in SpPLD was much longer than that in the several nucleases (PDB ID: 4GEL, 1BYR and 6EHI) (Figure 9b). Electrostatic surface potential showed that this loop was slightly negatively charged (Figure 9c).

C-terminal structure of SpPLD was also special.

**Figure 10:**
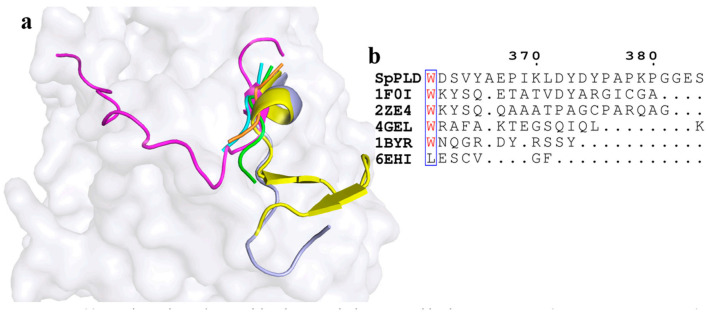
(**a**) Superimpostion and comparision the C-terminal structure with other PLDs. SpPLD (magentas, PDB ID: 7E0M), *Streptomyces* sp. PMF PLD (yellow, PDB ID: 1F0I), SaPLD (light blue, PDB ID: 2ZE4), Zuc (orange, PDB ID: 4GEL), NucT(cyans, PDB ID: 6EHI), Nuc (green, PDB ID: 1BYR). Two catalytic sites of SpPLD were shown in stick. (**b**) Sequence alignment of the amino acids that composed the C-terminal structure in SpPLD with other PLDs.

Superimpostion and comparision of the C-terminal structure of SpPLD with other PLDs were shown in Figure 10a. We found that the C-terminal loop was much longer than that of PLDPMF (PDB ID: 1F0I) and SaPLD (PDB ID: 2ZE4) (Figure 10b). Moreover, it flanked towards the catalytic pocket and was arranged as part of the catalytic pocket (Figure 10a). The shortest C-terminal was found in three nucleases (PDB ID: 4GEL 1BYR 6EHI). Polar contact analysis showed that there were no strong interations between the C-terminal loop and the neighbouring structure. However, these structural features were different from other PLDs (Figure 10).

## 3. Discussion

In the present study, the crystal structure of an extracellular PLD from *Serratia plymuthica* was characterized in detail. *Serratia plymuthica* is a plant-associated and plant-beneficial species that belonged to the family of *Enterobacteriaceae*. Members of the genus *Serratia* were ubiquitous in nature and their lifestyle varied from endophytic to free-living [22]. In agriculture, *S. plymuthica* was successfully used for the control of many soil-borne fungal pathogens of different crops (e.g., strawberry, rapeseed) [23,24]. *S. plymuthica* AS9 was of special interest because of its ability to stimulate rapeseed plant growth, to inhibit soil borne fungal pathogens and to increase oilseed production [22]. The complete genome sequence of this strain was available on NCBI databank [22]. As mentioned above, PLDs were ubiquitously distributed in various organisms and were also widespread in the plant kingdom [25]. In higher plants, the PLD family comprised of multiple members, and PLDs have been reported to play positive and/or negative roles in plant immunity, depending on the types of pathogens and the specific PLDs involved [26]. Roles of PLDs in plant-fungal interactions have been summarized by Li et al. [26]. In plants, PLDs hydrolyze membrane phospholipids to generate phosphatidic acids (PAs). Both PLDs and their lipid product PAs are involved in various physiological processes, including plant response to pathogens [26]. As the *Serratia plymuthica* is a plant beneficial species and functions to protect plants from fungal pathogens, we thus speculate that the extracelluar SpPLD from *Serratia plymuthica* AS9 may adopt a similar plant-like mechanism to play a defensive role. However, additional functional studies are required to understand its regulatory mechanism involved in biocontrol-related traits.

The structural characteristic of PLD superfamily, the HxKxxxxD (HKD) motifs, was presented in a single or double manner in the primary structure. The HKD motifs, in particular the histidine residue, were of critical importance in the catalysis, as confirmed by several studies [7,12,13,27,28]. Despite of the low sequence identity on the primary structure, their crystal structures showed a similar topology, which strongly suggested that all enzymes in the PLD superfamily shared similar structures. Moreover, the conservation of the catalytic motifs implied that the superfamily members had a similar structural core and directed for similar reaction mechanism towards a wide range of substrates. The catalytic mechanism of PLD enzymes was well characterized to proceed through a phosphohistidine intermediate that was formed after nucleophilic attack of the phospho-substrate by the first His residue of the HKD motif (H116 in SpPLD). The second His residue (H311 in SpPLD) activated a water molecule that attacked the phosphohistidine to release product and regenerated the free catalytic His residue 116 [1].

### 3.1. The Unique GS/GT Assembly Unit Found in SpPLD Made it Distinct from Other Members in the Same Family

Except for the HKD motif, a GG/S motif was also found conserved in the PLD superfamily. This motif located six residues downstream of the HKD motifs, in a close proximity to the catalytic histidines. The GG/GS motifs were suggested to maintain local conformation of the active site by positioning the catalytic His through the hydrogen bond network [1]. Two GG/S motifs were surrounded the putative active site in a symmetric manner. Ogino et al. proved that the conserved glycine-glycine (GG) and glycine-serine (GS) motifs, especially the Ser residue in *Streptoverticillium cinnamoneum* PLD (SciPLD) were essential in affecting transphosphatidylation activity. While each glycine residue in the GG/S motifs of *Stv. cinnamoneum* PLD was substituted by a serine residue, and the serine residue was also substituted by a glycine residue. It had been found that the G215S and G216S mutants exhibited approximately 9-and 16-folds higher transphosphatidylation activity, respectively, compared to the wild-type PLD. On the contrary, the S489G mutant showed reduced activity and G488S lost the activity completely. Accordingly, it was concluded that the serine residue in each motif was preferable for the PLD activity, though dispensable itself [29]. As for the SpPLD, the unique GS-GT motifs was first reported in the family. Structurally, threonine was similar with serine, there might be a mutations in evolution. Present constitution was quite similar with the nucleases (GS-GS). Even though, as there was still no data that supported the function of the GS-GT motif, especially on the transphosphatidylation activity, it still need further study.

### 3.2. Electronic Positive Extented Loop between α9 and β9 Made This Part Unsuitable for Binding the Acyl Chains Anymore

While superimpostion and comparision the structure of SpPLD with other PLDs within the same family, an extended loop between β9 and α9 was found distinct with other PLDs (Figure 7a). Moreover, electrostatic surface potential of SpPLD at this part showed net charge of +1, thus this loop was found positively charged. In constrast, the total net charge of the corresponding loop in SaPLD (2ZE4) was 0 and it showed hydrohobic character. In the *Streptomyces* PLD (1F0I and 2ZE4), the hydrophobic area (loop) was possibly used to accommodate the acyl chains [1]. However, for the SpPLD, a positive extended loop was found here. The positive area made this part unsuitable for binding the acyl chains. However, this part was quite similar with several nucleases (4ZEL 1BYR 6EHI) in the same family, where a positive charge was found. Detailed functions of this loop in SpPLD deserves further study.

### 3.3. The Shortened Loop Made SpPLD Unable to Form the Gate to Stable the Substrate

In SpPLD, a similar shortened loop existed between α10 and α11 in Nuc (1BYR) and Zuc (4GEL) (Figure 8a), compared to the large extend loop existed in PLDPMF (PDB ID: 1F0I) and SaPLD (PDB ID: 2ZE4). Recently, two flexible loops around residues Y126 and G381 forming a gate-like structure near the active site of SaPLD were pointed out as important factors in PL acceptance and/or fixation within the active site [30]. Moreover, while comparing the structures of the wild-type SaPLD and its inactive H168A mutant in complex with PL, a movement of the two loops for grasping the substrate upon binding was observed. Based on the results of the mutational studies and the structural data, a mechanism by which PLD recognized and binded to the PLs was proposed [1]. Because PLD acts on the PL-water interface, the enzyme needed to pull out one PL molecule from the aggregated substrate, and kept it within the active site to against the intrinsic property of spontaneous PL aggregation. In the following, the gate-like structure consisting of the loops around Y126 and G381 pulled one PL molecule into the catalytic pocket, and kept it fixed inside by closing the gate during catalysis. Although this concept was reasonable, it still required further verification [6]. As for the SpPLD, the lack of the gate made it hard to form the gate to lock the substrate. In constract, the composition at this part was quite similar with several nucleases (4ZEL 1BYR 6EHI) in the same family.

### 3.4. Loop Entrance Residues Composition Involved in Substrate Recognition Was Also Different from Other PLDs

Uesugi et al. investigated contribution of amino acid residues to the catalytic mechanism of *Streptomyces* PLD by generating chimeric PLDs from the highly homologous TH-2PLD and PLDP [31,32]. As a result, two loop regions (positioned between β7 and α7 and between β13 and β14) forming an entrance to the active site, were discovered to be important in catalysis and substrate recognition [31,32]. In the present SpPLD, the loop conformation corresponding to the two loops was quite similar with the *Streptomyces* PLD (1F0I and 2ZE9), which indicated that this loop may performed the same function in substrate recognition. However, results of sequence aligment of the amino acids that composed the loop between β11 and β12 with other PLDs indicated that there were still significant differences in amino acid composition. The influence of different amino acid composition of this loop on substrate recognition still needs further study.

### 3.5. A Unique C-Terminal Confromation Was Found in SpPLD

In SpPLD, a unique C-terminal conformation was found. From the present conformation, we were not able to find the tight contaction of the C-terminal with the neightbour site, which indicated that this part may not be stable in the conformation. However, because we find it flanked toward the catalytic pocket and was arranged as part of the catalytic pocket, it might function as the sensor to the substrate that substituted the function of shortened loop between helix α10 and α11. Further research is needed to illustrate the detailed function of this loop on the activity or selectivity of this enzyme by protein engineering.

## 4. Materials and Methods

### 4.1. Strains, Plasmids and Reagents

*Escherichia coli* DH5α and isopropyl β-D-1-thiogalactopyranoside (IPTG) were purchased from Takara Co., Ltd. (Dalian, China). SHuffle T7 Express competent *E. coli* cell was purchased from New England BioLabs (Beijing, China). pET-21a expression vector was purchased from Stratagene (La Jolla, CA, USA). Ni^2+^-nitrilotriacetate (Ni^2+^-NTA) affinity column, Sephadex G-25 Fine desalting column and Superdex-200 gel filtration column were obtained from GE Healthcare Life Sciences (Pittsburgh, PA, USA). Bradford Protein Assay Kit was obtained from Shanghai Sangon Biological Engineering Technology (Shanghai, China). The eleven Crystallization Kits (crystal screen 1 and 2 (HR2-130), Wizard Class 1 and 2 and 3 and 4, SaltRx (HR2-107, HR2-109, HR2-136), Index (HR2-134) and Stock Options pH (HR2-241)) were all purchased from Hampton research company (Aliso Viejo, California, USA)). All other chemicals used in the present study were analytical grade.

### 4.2. Bioinformatic Analysis of SpPLD

The SpPLD protein from *Serratia plymuthica* AS9 that expressed in this study was deposited in the NCBI-Protein databases under the accession number of AEF43698.1. The basic physical and chemical properties of SpPLD were analyzed using ProtParam online tools (https://web.expasy.org/protparam/) (accessed on 5 February 2021). The signal peptide of SpPLD was predicted using online tools SignaIP v. 5.0 web server (http://www.cbs.dtu.dk/services/SignalP/) (accessed on 5 February 2021) [33]. Structure-based multiple amino acid sequence alignment was performed with T-Coffee servers (http://tcoffee.crg.cat/) (accessed on 5 February 2021) [34] and the ESPript 3.0 web server (http://espript.ibcp.fr/ESPript/cgi-bin/ESPript.cgi) (accessed on 5 February 2021) [35]. Protein sequence similarity and identity between SpPLD and other PLDs were analyzed with BLASTP (https://blast.ncbi.nlm.nih.gov/Blast.cgi) (accessed on 5 February 2021). Phylogenetic analysis by the neighbor-joining method with 1000 bootstraps was created by the MEGA 7.0 program [36]. The GenBank accession numbers or PDB ID of the PLD sequences for multiple sequence alignment and constructing the phylogenetic tree were given in the Figure 2 and Figure 3. A structure-similarity search for SpPLD was performed by using the DALI online server [37].

### 4.3. Protein Expression and Purification

The gene encoding the mature protein of SpPLD without the signal peptide and containing 8× his-tag at the N-terminus was artificially synthesized by Sangon Biotech, Inc. (Shanghai, China) and inserted into the pET-21a expression vector to form pET21a-(His)8-SpPLD and transformed into *E. coli* DH5α. Plasmids were confirmed by sequencing. The constructed vector was further transformed into Shuffle T7 express competent *E. coli*. To purify SpPLD, Shuffle T7 express competent *E. coli* cells bearing recombinant expression plasmid pET21a-(His)8-SpPLD was cultured overnight at 37 °C in 5 mL Luria-Bertani (LB) medium containing 100 μg·mL^−1^ ampicillin. Next, 2000 mL of LB medium in a shake flask, supplemented with the same antibiotics, was inoculated with 2 mL of the overnight culture and incubated at 37 °C. When the cell density at A_600nm_ (OD_600nm_) reached 0.6–0.8, IPTG was added to the cultures to the final concentration of 0.2 mM. Culturing continued for 20 h at 16 °C. Then, the cells were collected by centrifugation at 5000× *g* for 8 min at 4 °C. The harvested cells were resuspended and sonicated in lysis buffer (50 mM Tris, 500 mM NaCl and 10 mM imidazole, pH 8.0) and then centrifuged at 10,000× *g* for 30 min at 4 °C. Finally, the recombinant target protein was purified by a Ni^2+^-NTA packed columns and washed with buffer A (50 mM Tris-HCl, 500 mM NaCl, 40 mM imidazole, pH 8.0). The target proteins were eluted with buffer B (50 mM Tris-HCl, 500 mM NaCl, 200 mM imidazole, pH 8.0). The fractions containing the proteins were then loaded onto the desalting column Sephadex G-25 and washed with buffer C (50 mM Tris-HCl, 500 mM NaCl, pH 8.0). Gel filtration chromatography was further performed with the AKTA purifier protein purification system (GE Healthcare Life Sciences, Pittsburgh, PA, USA) using Superdex-200 gel filtration column and washed with buffer D (20 mM Tris-HCl, 100 mM NaCl, pH 8.0). For selenomethionine (SeMet)-derived protein expression, the protocol was similar to the above except the medium according to the method reprted by Hendrickson et al. [38]. 30 KDa Ultrafiltration tube was used to concentrate protein. The purity and molecular mass of SpPLD were analyzed by 12% SDS-polyacrylamide gel electrophoresis (SDS-PAGE). Protein concentration was determined with Bradford Protein Assay Kit.

### 4.4. Crystallization and Structure Determination

Preliminary screening for crystallization conditions of SpPLD was carried out using the the hanging drop technique at 16 °C. Conditions were surveyed using eleven commercial screening kits by mixing 1 µL of 10 mg/mL protein and 1 µL of reservoir solution in a 24-well plate. After 3 days, these growing crystals were usually flake and fragile, not suitable for X-ray diffraction. Therefore, a series of two factor variable orthogonal experiments were performed to optimize the precipitant concentration and the pH of crystallization reagent. Eventually, high-quality monocrystal of SpPLD was obtained under the condition of 11% PEG 8000, 8% Ethylene glycol, and 0.1 M HEPE/sodium hydroxide pH 7.6. Similarly, high-quality crystal of SeMet-derived SpPLD was obtained under the condition of 1.2 M Diammonium hydrogen phosphate, 0.2 M Sodium chloride, 0.1 M Imidazole/hydrochloric acid pH 7.8. crystals of SpPLD and SeMet-derived SpPLD used for X-ray diffraction were rapidly soaked in reservoir solution with 25%, 30% glycerol respectively as cryo-protectant, mounted on loops, and then flash-frozen in a 100 K nitrogen gas stream. During the data collection, the crystal was kept at 100 K. Diffraction images were collected at beam line 18U1 of the Shanghai Synchrotron Radiation Facility (SSRF, Shanghai, China).

Diffraction data were processed using the XDS package [39]. Manual model building was performed with COOT [40] and used SeMet-derived protein crystals to determination the phase for the molecular replacement method by the program PHASER in the CCP4 program suite [41]. The crystallographic refinements were executed using PHENIX, and the qualities of the final model were evaluated by PROCHECK [42,43]. Finally, the coordinates and structural factors of SpPLD were deposited in the Protein Data Bank (PDB) with PDB ID: 7E0M. Diagrams of the protein structure were drawn by PyMOL software [44].

## 5. Conclusions

PLDs are now recognized as one kind of important tool for the enzymatic synthesis of PLs. The important application values of PLDs have been awaited for a long time. Even with the deepening understanding of the PLD superfamily, in spite of the PLDs sharing common folds and catalytic mechanisms involving two His residues, the information is far from sufficient to be used as a screening criterion yet, especially concerning the substrate selectivity and transphosphatidylation activity. Therefore, getting more illumination of the PLD structures and structure–function relationships is particularly important. In the present study, we reported the crystal structure of SpPLD and analyzed the structural differences of PLDs from different microbial sources. Although the two HKD and two GG/S motifs existed in the protein which indicated that it belonged to the superfamily, still more questions remain to be answered, such as those concerning the substrate selectivity. Furthermore, because the SpPLD has shown some structural properties which are the same as the nucleases, there is the question ofwhether it has the nucleic acid hydrolytic activity? Additionally, what are the functions of the different loop conformations in the catalytic pocket? Elucidation of these issues would help to understand the catalytic mechanism in more details. The combination of extensive mutational analysis and crystal structure determinations will shed light on it. Even so, the present study further enriched the information of phospholipase structure databases and laid an important foundation for the downstream enzyme screening and modification.

## Figures and Tables

**Table 1:** Structural information of resolved phospholipases D (PLDs) in the PLDc 2 (PF13091) family.

No.	Taxonomy	Name	Organism	PDB Codes	Deposition Date	Reference
1	Microorganism	Nuc	*Salmonella typhimurium*	1BYS (complexed with Tungstate) 1BYR	1998	[7]
2	Microorganism	PLDPMF	*Streptomyces* sp. PMF	1F0I (apo) 1V0R (Tungstate-inhibited form) 1V0S (Uninhibited form) 1V0T (complex with product glycerophosphate) 1V0U (complex with product glycerophosphate) 1V0V (complex with substrate dibutyrylphosphatidylcholine) 1V0W (complex with substrate dibutyrylphosphatidylcholine) 1V0Y (complex with substrate dibutyrylphosphatidylcholine)	2000 2004	[12,13]
3	Microorganism	BfiI	*Bacillus firmus*	2C1L 3ZI5 (C-Terminal Fragment of BfiI domain in complex with cognate DNA)	2005 2013	[10,19]
4	Microorganism	SaPLD	*Streptomyces antibioticus*	2ZE4 2ZE9 (H168A mutant complex with phosphatidylcholine)	2007	[20]
5	Mammal	mZuc / PLD6 / MitoPLD	*Mus musculus*	4GGJ 4GGK (complex with tungstate)	2012	[9]
6	Arthropoda	Zuc	*Drosophila melanogaster*	4GEL 4GEN (monomer) 4GEM (K171A mutant) 4H4A (C-terminal domain)	2012	[21]
7	Microorganism	NucT	*Helicobacter pylori*	6EHI	2017	[11]
8	Mammal	hPLD1	*Homo sapiens*	6U8Z 6OHR (complex with compound 5)	2019	[18]
9	Mammal	hPLD2	*Homo sapiens*	6OHM (complex with tungstate) 6OHO6OHP (complex with halopemide) 6OHQ (complex with compound 4) 6OHS (complex with compound ML299)	2019	[17]
10	Microorganism	SpPLD	*Serratia plymuthica strain* AS9	7E0M	2021	Present research

**Table 2:** Data collection and refinement statistics.

	SpPLD
Wavelength	
Resolution range	23.88–1.79 (1.854–1.79)
Space group	P2_1_2_1_2_1_
Unit cell	a = 78.78Å, b = 97.57 Å, c = 117.03 Å, α = 90 Å, β = 90 Å, γ = 90 Å
Total reflections	928,590 (56,570)
Unique reflections	79,256 (6680)
Multiplicity	11.7 (8.4)
Completeness (%)	96.98 (79.33)
Mean I/sigma(I)	12.77 (1.47)
Wilson B-factor	25.38
R-merge	0.1337 (1.706)
R-meas	0.1397 (1.822)
R-pim	0.0396 (0.6073)
CC1/2	0.997 (0.503)
CC*	0.999 (0.818)
Reflections used in refinement	82,957 (6676)
Reflections used for R-free	4150 (335)
R-work	0.1695 (0.2706)
R-free	0.1848 (0.3129)
CC(work)	0.969 (0.788)
CC(free)	0.967 (0.713)
Number of non-hydrogen atoms	6902
macromolecules	6104
ligands	5
solvent	793
Protein residues	772
RMS(bonds)	0.011
RMS(angles)	1.47
Ramachandran favored (%)	97.14
Ramachandran allowed (%)	2.86
Ramachandran outliers (%)	0.00
Rotamer outliers (%)	0.93
Clashscore	4.92
Average B-factor	30.06
macromolecules	28.77
ligands	38.60
solvent	39.87

Statistics for the highest-resolution shell are shown in parentheses.
